# Going Abroad

**Published:** 1873-05

**Authors:** 


### HALL'S

### JOURNAL OF HEALTH.

Vol. XX.—MAY, 1873.—No. V.

### GOING ABROAD.

The Atlantic may be crossed at various prices, from sixty-five dollars to a hundred and thirty, in gold. The Anchor line is the cheapest; the White Star line the newest, best; the Cunarders the safest. Never buy a return ticket for the slight savingof ten or fifteen dollars. Leave yourself free to come back home when and how you please; better return by a different route or a different line—then you will know more.

Whether man or woman, make two trunks do you: a large one to be stowed away until you get on the other side; a small one, not over twelve inches deep, so that you can put it under your state-room berth—this is to contain the indispensable things of your voyage.

When you go aboard the vessel dress in your commonest clothing, for in a few hours you will be "casting up," and possibly find yourself sprawling on the deck.

Put on thick winter clothing, even if the thermometer is at a hundred degrees, and you seem to yourself to be almost melting with heat. But nobody will follow this advice who has never been to sea; those who have, know its importance. Let all your clothing be plain—such as will wear well; then you won't be afraid of hurting it, and will be left free to enjoy yourself. Life on shipboard is ruinous to good clothing; however good it may have been, it is unfit to wear after you land; the cleanest ship that floats is a filthy habitation; such clothing should be left at the hotel, to be worn on the return voyage. Thick-soled boots and shoes are indispensable while aboard, and also some outer garment which will turn off the rain. If our wives and daughters could only be persuaded of the fact, that the better class of people should always dress plain for out-doors, and that they take it for granted that travelers who dress plain can afford to do so, an immeasurable amount of annoyance and discomfort would be escaped. One feels so much easier in his common home dress, that it is a wonder that so much comfort is sacrificed in the wish to impress strangers with the fact that you are magnificent and rich and all that. Foreigners understand that you are travelers, and do not expect you to wear a wedding dress every time you step on the pavement. Even if you are invited out, you are readily excused from dressing as if for a ball. Do have some sturdy republican independence when you reach the other side; but remember that boorishness is not independence. The loud talk, the boisterous laugh, the slap on the shoulder, the "hale fellow, well met," idea, a contempt for the conventionalities of cultivated society,—none of these have the true ring of independence.

Above all things, don't brag; it only exposes you to the irredeemable contempt of all well bred foreigners; if you wish to ingratiate them into your favor, have the courtesy to speak well of all you see, if it is merited; if manners and customs which present themselves to you are antagonistic of your views and experiences, wait for further observation, and you may find out later that difference of mode of thought, of climate and country, may greatly modify your opinions. Don't volunteer information about yourself, your family, your acquaintances, or your fortune. You may think a million is a great deal; the person to whom you may be talking is possibly worth fifty, and ten to one is dressed more plainly than yourself. Jonathan is so used to going ahead and blurt out all he knows, and a good deal more sometimes, that he would do well to practice reticence and retiracy under the directions of some competent private teacher. Be sought out rather than seek, and don't make yourself too common.

Hundreds of thousands of dollars are squandered annually by American travelers in the foolish pride of getting the best seats. It is a common saying in England, that fools and Americans ride in first-class cars.

A British nobleman was one day securing a seat by rail for himself and body servant, for whom he took a first-class ticket, and a second-class for him. "Why, my lord," said the agent, "do you travel in the second-class train?" "Because there is no third."

A man should have only two suits: one for common use, stout, strong and warm; another of precisely the same kind to wear "on occasion." A lady should have only one good black silk dress, for rare cases, and another suit which does not show dirt, strong and warm. With these, persons can travel all over Europe and be respected at the tables of the first people of the realm. It is the manner, not the dress, which secures and maintains the respect of persons of position. Hundreds of dollars will be saved if, when you reach the continent, you will leave your American trunks and provide yourself with willow baskets. An empty trunk will weigh as much as a basket and its fill of traveling articles; every pound of luggage is an extra charge.

Be ashamed to cheat your country out of a few dollars of custom dues, by endeavoring to smuggle home dutiable articles in the privacy of your portmanteau. If you must bring home curiosities and all sorts of traps for yourself and forty friends, box them up, marking the contents; send them as freight, and be done with it. The worry and apprehension experienced by many on the point of landing, and even for days beforehand; the hours and hours spent in framing subterfuges, and the fear of discovery, are unmistakably degrading. You know you are doing a mean thing, and you had better far toss all your trip-traps overboard.

There is a rich banker in New York who took passage in a Cunarder from Liverpool with his beautiful young wife. It is positively forbidden to ship a dog at any price; but she must have her poodle on the same ship. It was carried aboard in a carpet-bag. Food was taken from the table at every meal and carried to the state-room for the dog. And what a time they had, every now and then, to keep the little brute from yawping. The steward was paid. But before the ocean was half crossed an officer comprehended the situation. It was too late to turn the ship back, and it was hardly worth while to throw the thing overboard; and what fun it was for them all to witness, day after day, the little stealings and fibbings and hidings of man and wife. The day before the vessel landed in New York a bill was handed to the banker of one hundred and twenty dollars in gold, for "poodle's passage." It was paid without a word; of course it was. Any objurgation in that direction would have elicited very inconvenient elucidations and revelations. If you don't want to find yourself in a foreign land some sunny morning without a dollar, get a letter of credit from some old house, even if you have to pay a little more. Brown Brothers are good Presbyterians, and are of course safe; then there are Drexel & Morgan, Winslow, Lanier & Co., Munroe & Co., and others, but the above are as solid and as safe as the deepest foundations of the cordilleras.

By all means apply for your passport a month beforehand, and take good care of it; it will be a good friend to you in various emergencies.

Of course you want to know something when you come back. But you should know beforehand what you ought to know. Then get

HARPER'S HANDBOOK

of Foreign Travel; a new edition of it is got up every year, in order to make it reliable and to have it up to the time. Study it well while on your outward bound voyage; read it with a great deal of care, and remember what you read. It tells you at what hotels to stop, what are the charges of many of them, what it will be most interesting to see at all places of prominence; in short, it is invaluable.

As to the routes of travel each one knows his own circumstances best, the objects he wishes to accomplish, and the extent of his purse and travels. One thing, however, is worthy of consideration; it is better to see a single one-horse village well, and become acquainted with all its distinguishing features, so as to be able to tell all about it, ten or fifty years hence, than to travel all over Europe, and be only able to say of London, "it was horrid, with its everlasting fog and daily rain and pesty coal dust and smut and smoke;" or of Paris, that "it was splendid;" of Heidelberg, "it was queer;" and of Unter den Linden, "it was grand." Information of this kind is not only tantalizing to the listener, it is senseless, it is stupid. Two routes only will be named.

Leave New York for Queenstown, Ireland; travel by way of Dublin north to the Giant's Causeway, one day from Glasgow, which is six hours from Edinburgh and Abbotsfond; thence by steamship to Rotterdam at the mouth of the Rhine in a day or two; then go up the Rhine to Manheim; this requires several days, as the steamers do not travel at night; this gives an opportunity of seeing the famous old river by daylight along its whole navigable extent. Heidelberg is half an hour inland from Manheim; from this point travelers can diverge to all points of the compass.

Another route is to take passage direct to Glasgow from New York by the Anchor line of steamers; fare, seventy dollars in gold. Some prefer to go direct to London from New York, *via* Liverpool; others direct to Havre, $1[illegible]0. There is a line of steamers direct from New York to the mouth of the Rhine at Rotterdam. In returning home, glorious old England can be seen at leisure. It will be more interesting to see the largest places last.

Three Boston sisters were met, a year ago, in Switzerland; they had been from home three years; they dressed very plainly, but their clothing was good, well-made and appropriate; they put up at good places, made no noise, no show, attracted no attention; but wherever they were, they would make themselves comfortable; they spent no idle time; they would study "Harper's" and other guide-books while in the house; hurry off to the places indicated; look at them, book in hand, and when they came back, would read the books over again and compare facts with statements. They did not seem to be in a hurry to get home, and thought they might stay a year or two longer. These girls will know something when they come back; they will be able to tell of what they have seen, and will be interesting conversationists in any company; this is the true method of travel. There is an incalculable store of satisfaction for them in the future, in talking of the places they have visited with persons who have also seen them, and always hereafter, when they take up a paper having the name or a description of a place they have seen, pleasant memories and satisfactory associations will come trooping after.

